# Anterior Cutaneous Nerve Entrapment Syndrome: An Underestimated Diagnosis

**DOI:** 10.7759/cureus.48165

**Published:** 2023-11-02

**Authors:** Buse Cagla Ari, Elifnaz Sahin

**Affiliations:** 1 Neurology, Bahcesehir University Medical Faculty, Istanbul, TUR

**Keywords:** gabapentin neuro, treatment choices, aterior cutaneous nerve entrapment syndrome acnes, anterior cutaneous nerve entrapment syndrome, chronic abdominal wall pain

## Abstract

Introduction: In the process of differential diagnosis concerning chronic abdominal wall pain (CAWP), several conditions are typically considered, including abdominal wall hernias, endometriosis, thoracic nerve radiculopathies, xiphoidalgia, and lower rib pain syndromes. Notwithstanding these, there exists an additional condition that is often overlooked initially: anterior cutaneous nerve entrapment syndrome (ACNES). This syndrome is characterized by the entrapment of cutaneous nerve branches responsible for supplying the abdominal wall. The diagnostic procedure for this condition can present notable challenges.

Case presentation: The subject of concern was a male patient aged 30, who presented with persistent CAWP. Despite conducting comprehensive analyses of his blood, urine, and imaging studies, no anomalies were detected. However, he exhibited positive results for the pinch test and Carnett's sign. Based on the outcomes of his clinical assessment, the patient received a diagnosis of ACNES. Subsequent administration of gabapentin resulted in a notable alleviation of his symptoms.

Conclusions: This case report highlights the referral of a patient to a neurology clinic owing to abdominal wall pain. Given the rarity of ACNES reports, our objective was to delineate the findings of our patient, with the aim of augmenting clinicians' understanding of this condition.

## Introduction

Chronic abdominal wall pain (CAWP) manifests as discomfort occurring between the diaphragm and the upper plane of the pelvic cavity. Incidence estimates indicate that approximately one in 1800 individuals experience abdominal wall pain [1]. When undertaking differential diagnosis, considerations encompass abdominal wall hernias, abdominal wall endometriosis, thoracic nerve radiculopathies, xiphoidalgia, and lower rib pain syndromes. Notably, after obtaining normal results from blood analysis, urine testing, and imaging workup, an additional differential diagnosis to be considered is the frequently overlooked anterior cutaneous nerve entrapment syndrome (ACNES), wherein the primary etiology involves the entrapment of cutaneous nerve branches supplying the abdominal wall [1,2]. Reported statistics reveal that a mere 2% of patients with acute abdominal wall pain receive a diagnosis of ACNES. Hence, recognition, diagnosis, and treatment of this condition are imperative for ensuring patient comfort and enhancing overall quality of life [1]. In this context, we present the case of an individual who endured abdominal wall pain for a significant duration and was eventually diagnosed with ACNES, a condition that proved challenging to identify.

## Case presentation

A 30-year-old male patient presented persistent abdominal pain and a burning sensation lasting two months. The pain initiated gradually in the left inguinal region, diffused throughout the abdomen, and ultimately localized near the umbilicus in subsequent weeks. Initial examination by a urologist included a complete blood count, urinalysis, and pelvic MRI. A diagnosis of acute cystitis prompted the administration of ciprofloxacin 500 mg twice daily for seven days. As symptom resolution was not achieved, referral to the General Surgery Department followed, with subsequent evaluation via complete abdominal USG and chest and abdominal CT scan, revealing no definitive diagnosis. Further investigations, including endoscopy and colonoscopy, yielded normal results, leaving the etiology of his symptoms elusive. Notably, the patient had no pertinent medical history, prior surgeries, ongoing medication use, or excessive alcohol consumption. Given the persistent pain, a neurologist consultation was advised. Neurological assessment unveiled hyperesthesia at the left umbilical region, with evident protective responses during palpation. Positive outcomes were observed with both the pinch test and Carnett's sign. A repeated analysis of laboratory and urinary parameters returned within normal limits. Based on the cumulative examination findings, the patient received a diagnosis of ACNES and was subsequently treated with gabapentin at a dose of 300 mg three times a day, along with vitamin B complex (B1, B6, and B12) and 600 mg of alpha-lipoic acid daily. Following three weeks of treatment, a notable reduction in symptoms and an enhancement in the patient's quality of life were observed. The patient remains under ongoing observation without any reported complaints to date.

## Discussion

The estimated prevalence of abdominal wall pain is approximately 1 in 1800 worldwide. Females are four times more prone to ACNES compared to males. Although cases have been reported in both pediatric and geriatric populations, two distinct incidence peaks have been documented, occurring in the 15-20 and 35-45 age ranges [3]. The purpose of composing this case report is to underscore the significance of considering the exclusionary diagnosis of ACNES. An elaborate patient history and comprehensive physical examination constitute the primary approach toward diagnosis. CAWP stands as the principal symptom of this condition, characterized by varying patterns in terms of duration, attributes, and intensity. Pain presentation can manifest acutely, transitioning into a persistent dull ache and exhibiting a fluctuating severity ranging from mild to severe [2]. The transmission of sensory data from the skin of the abdominal wall is facilitated by the intercostal nerves, which are situated amid the internal oblique, and transversus abdominis muscles. Specifically, anterior cutaneous intercostal nerve branches (Th8-12) traverse from five discrete points positioned medially to the linea semilunaris, entering the rectus abdominis muscle posteriorly at a perpendicular angle, consequently contributing to the formation of the clinical presentation's trigger points [4]. The trajectory of ACNES can be delineated as the manifestation of localized neuropathic pain resulting from the entrapment of the cutaneous nerve branches responsible for innervating the abdominal wall. These nerve branches extend anteriorly and traverse a fibrous ring within the posterior sheath of the rectus abdominis muscle. Encased within an adipose-enriched neurovascular bundle, these cutaneous nerves are increasingly vulnerable to external influences as adipose tissue degenerates. Prolonged mechanical stress at these pivotal junctures may precipitate the onset of chronic pain [5]. While pain serves as a defining characteristic of ACNES, it is notable that around 50% of patients concurrently encounter various visceral manifestations. Among these, abdominal bloating, nausea, and altered defecation stand out as the most commonly reported visceral symptoms [6]. Considering this insufficient knowledge, ACNES must be approached with increased awareness.

Further validation of the diagnosis is attainable through the utilization of Carnett's test [7]. The examination entails the application of pressure by the examiner on the area of utmost sensitivity, while the muscles of the anterior abdominal wall are contracted. Ordinarily, the discomfort intensifies over time (indicative of a positive Carnett's test); however, occasionally, the aggravation of pain is not more pronounced than that experienced during muscle relaxation. Conversely, individuals experiencing pain stemming from the abdominal viscera typically experience diminished discomfort upon tensing the muscles of the abdominal wall (signifying a negative Carnett's test). Adequate voluntary contraction of the anterior abdominal muscles is imperative for the proper execution of this assessment [2,8]. In the context of this clinical syndrome, van Assen et al. relied upon a compilation of signs and symptoms established by a software program, which has derived its foundation from the systematic documentation of patient consultations. The documented constellation of signs and symptoms includes the presentation of abdominal pain during emergency department visits, the manifestation of abdominal tenderness featuring a small trigger point (<2 cm2) delineated laterally by the rectus abdominis muscle, absence of anomalies detected during laboratory analyses and imaging procedures, and the alleviation of symptoms after an injection of 5-10 mL of 1% lidocaine [1].

Recognizing the ailment, prioritizing symptom alleviation, and imposing limitations on strenuous activities involving the abdominal wall musculature constitute the treatment objectives. Providing reassurance to patients afflicted with CAWP, despite the potentially distressing and debilitating nature of the symptoms, that the condition typically lacks progressive characteristics and does not engender enduring health implications represents the foremost and arguably pivotal measure in their therapeutic regimen [5,9]. After conservative treatment, the subsequent course of action for patients experiencing mild to moderate pain entails trigger point injections, serving as both a therapeutic and diagnostic intervention. These injections are typically delivered as a composite therapy, incorporating a local anesthetic in conjunction with a glucocorticoid or as a standalone anesthetic agent. At an average follow-up duration of 13.8 months, a notable 78% of patients exhibited indications of a persistent positive response to the administered injection therapy [10,11]. In the event of an inadequate response to the aforementioned therapeutic interventions, a sequential approach should be adopted. Consideration may be given to the assessment of chemical neurolysis, pulsed radiofrequency, and surgical interventions such as decompression of entrapped nerves or intraperitoneal onlay mesh reinforcement in accordance with the individual patient's requirements. Notably, a study documented a 61% success rate at the 32-month mark subsequent to the surgical procedure (anterior neurectomy) among patients subjected to long-term follow-up [9,10,12]. Furthermore, nonsteroidal anti-inflammatory drugs, antiepileptic agents, and low-dose tricyclic antidepressants are viable options within the spectrum of systemic therapy aimed at managing the patient's symptoms [5].

## Conclusions

ACNES represents a condition that significantly compromises patients' functional capacity and overall quality of life, warranting consideration in the spectrum of differential diagnoses for CAWP. Implementation of neuropathic pain management strategies holds promise in mitigating the patient's distressing symptoms.
